# Alexithymia Increases the Headache Pain Index in Women with Migraine: Preliminary Results

**DOI:** 10.3390/jcm14051629

**Published:** 2025-02-27

**Authors:** Eugenia Rota, Elisa Cavagnetto, Paolo Immovilli, Nicola Morelli, Pavel Salari, Alessandro Battaggia

**Affiliations:** 1 The Neurology Unit, San Giacomo Hospital, 00186 Novi Ligure, Italy; eugenia.rota.md@gmail.com (E.R.); eli.cavagnetto@gmail.com (E.C.);; 2 The Neurology Unit, Guglielmo da Saliceto Hospital, 29121 Piacenza, Italy; 3 The Neuroradiology Unit, Guglielmo da Saliceto Hospital, 29121 Piacenza, Italy; nicola.morelli.md@gmail.com; 4 SVEMG–Scuola Veneta di Medicina Generale, 35129 Padova, Italy; a.battaggia@libero.it

**Keywords:** alexithymia, migraine, headache, pain, disability

## Abstract

**Background:** Alexithymia is characterized by a deficit in identifying and communicating feelings. Emerging evidence suggests that it is highly prevalent in migraine, where it could affect the pain expression. This pilot study on female migraineurs aimed at assessing any relationship between alexithymia and headache attacks in terms of frequency and pain intensity. **Methods**: All the patients (42) who fulfilled the diagnostic criteria for migraine were enrolled in this pilot, observational, cross-sectional study after having obtained written informed consent. A psychological assessment was made of each patient to identify any alexithymia using the TAS-20 scale, for anxiety/mood comorbidity (the STAI-Y1, STAI-Y2, and BDI-II) and for migraine-related disability (the HIT-6). An HPI index (attack frequency x pain intensity) was also calculated for each patient, based on their headache diaries. A multivariate analysis was performed to investigate any association among the TAS-20 score, HPI score, and the following covariates: BDI-II, STAI-Y1, STAI-Y2, HIT-6 scores, age, education, and disease duration. **Results**: Overall, 35.6% of the sample were given a diagnosis of alexithymia. After removing a subgroup of 7 subjects with HPI > 100, with more severe psychiatric comorbidity and a longer disease duration from the whole sample, a multivariate analysis detected a statistically significant (*p* = 0.010) association between the HPI and TAS-20 scores. **Conclusions**: This pilot study suggests that alexithymia may play a role in increasing the frequency and pain intensity of migraine attacks, consequently worsening disability in female migraineurs. Further studies are required to confirm this finding.

## 1. Introduction

Alexithymia (literally, “no words for feelings”, a term first coined by Sifneos in 1973 [1]) is a multidimensional psychological construct, which refers to an impaired ability to identify and communicate feelings and to distinguish between feelings and bodily sensations [2].

It has been reported that it is highly prevalent in chronic pain disorders and that it may affect the clinical phenotype of the pain, entailing deficits in emotion-regulating systems [3,4,5]. This psychological trait has numerous consequences, including misleading affected individuals to interpret the somatic manifestations of emotions as signs of disease [2].

Interestingly, alexithymia is emerging as a potential characteristic trait of both episodic and chronic migraine, regardless of the disease severity [6,7]. Moreover, it seems to be related to medication overuse, trauma, and/or stressful events in chronic migraine [8]. In fact, it has long been known that migraineurs often have particular personality profiles and psychological traits that may well contribute to migraine onset and chronification and lead to medication overuse [9,10].

Other studies have reported a complex interplay between alexithymia and psychiatric comorbidity in migraine, where it may coexist with depression and anxiety in migraineurs [11,12,13]. Indeed, migraine is often comorbid with psychiatric disorders, such as depression and anxiety, with a bidirectional relationship [14].

Consequently, the question arises as to whether the psychological trait of alexithymia plays a role in the clinical expression of migraine or whether the relationship between alexithymia and this primary headache is mediated by the psychiatric comorbidity.

According to the former hypothesis, we believe that a novel therapeutic approach targeting alexithymia could be justified and potentially beneficial for migraine prophylaxis.

This pilot study aimed at assessing any relationship between alexithymia and headache attacks, in terms of frequency and pain intensity, in female migraineurs, either with aura or without. Both the frequency and intensity of pain are the pivotal determinants of the pain phenotype in migraine attacks and the main factors responsible for the disability that arises due to migraine pathology. So as to rule out the potential influence of the psychiatric comorbidity on the relationship between alexithymia and headache severity, anxiety and depression were taken into account in the study sample.

## 2. Materials and Methods

This is a pilot, observational, cross-sectional study. All the female patients, aged 18–70, who consecutively referred to the Headache Center of Novi Ligure, Alessandria, Italy, for a first visit within a two-month period were enrolled in this study if they fulfilled the ICHD-III [15] diagnostic criteria for either episodic or chronic migraine (with or without aura) after having given written informed consent. This study was approved by the hospital’s Institution’s Ethics Committee (Protocol number: Asl20.Neuro.20.01).

Exclusion criteria were as follows: impaired ability to provide a detailed history due to a language barrier, an ongoing major depressive episode, relevant medical or surgical comorbidities (a surgical intervention in the previous year), an ongoing migraine attack, having taken painkillers for an acute attack in the previous 72 h, and/or lack of written informed consent.

A semistructured interview was given to each patient where demographic data were collected, that is, age, gender, school years, and migraine duration, and a psychological assessment was also made.

The Toronto Alexithymia Scale (TAS-20) scale [16] was used to assess alexithymia.

A TAS-20 score of >60 was considered consistent with a diagnosis of definite alexithymia, while a TAS-20 score between 51 and 60 indicated a borderline degree of alexithymia [16].

The State-Trait Anxiety Inventory (STAI)-Y1 and -Y2 (score 20–80 each scale) [17] was used to assess state and trait anxiety, respectively; the Beck’s Depression Inventory (BDI)-II (score 1–63) [18] was used to assess depression; and the Headache Impact Test (HIT)-6 (score 36–78) [19] was used to assess migraine-related disability.

All patients were given a diary for the day-to-day recording of the severity and duration (in hours) of their headaches and intake of any analgesics (by type), which they filled in for a period of 1 month. The headache pain intensity was coded according to the Numerical Rating Scale (NRS) [20] as a number between 0 (no pain) and 10 (the worst imaginable pain).

A Headache Pain Index (HPI) was calculated, as reported in previous studies [21]: intensity × frequency, where intensity was the sum of the intensity of the daily pain in a month divided by the number of days with pain and frequency was the number of days with pain in a month.

Statistical analysis: the patients’ demographic and clinical characteristics were determined by a descriptive analysis based on the average and standard deviation (S.D.) values, as well as the median and interquartile range (I.Q.R.) when appropriate. Whenever necessary, the assumption of normality for the distribution of variables was tested by the Shapiro–Wilk and Shapiro–Francia parametric hypothesis test of composite normality.

A multivariate analysis assessed any association between the HPI score (dependent variable) and the TAS-20 score (primary independent variable), adjusting for the following covariates: demographic (age, marital status, and scholarity in years) and clinical status (disease duration, monthly migraine days, BDI-II, STAI-Y1, STAI-Y2, and HIT-6 scores).

The observation of a skewed distribution of the values of the dependent variable (HPI) guided the choice of a GLM model (Family = Gaussian, link = Identity), where the outcome was expressed as a logarithm (LN-HPI). The model predictions were then converted into natural units, applying the method described by Cameron and Trivedi [22].

The strength of the association between the covariates and the endpoint was initially studied by a monovariate approach; that is, the selection of clinically relevant variables for patients to be evaluated by multivariate analysis (initial plethoric model) was made using inferential criteria with a generous *p* value cut-off (*p* value < 0.20) [23].

The further selection of covariates in the transition from the initial redundant model to the final parsimonious model was made by adopting the usual confidence levels (the statistical significance was set at 0.05).

The presence of interactions between the variables was investigated so as to study any effect modifiers. Whenever necessary, the variables were modeled in the most suitable way to match the mathematical relationship with the endpoint. It was ensured that the final model respected the mathematical-statistical assumptions using the Pregibon test so as to verify the correct specification; the Breusch-Pagan/Cook-Weisberg test assessed the homoskedasticity of the residuals, the identification of influential points was performed via the “DFBETA statistic”, and their importance was studied by sensitivity analysis in models with and without “influential” observations.

The selection of variables, the assembly of the final model, and the postestimation tests were performed separately in two models, that is, (a) a model including all observations and (b) a model that included only patients with an HPI score of ≤100. Indeed, there was a strong bimodality in the distribution of HP1 values in the total sample, and 7 patients with an HPI of >100 appeared to be a clinically identifiable subgroup in as much as they had peculiar characteristics that differed from the rest of the sample. This allowed us to remove from the final statistical model this subgroup of patients and to repeat the analyses on patients with an HPI score of ≤100. This choice will be illustrated and explained better in the following paragraph (Section 3).

Access to all the data in the statistical analysis is available on request.

Data analyses were carried out by the Stata/MP 17.0 -6 score, StataCorp LLC 4905.

Lakeway Drive, College Station, TX 7945, USA.

## 3. Results

This study enrolled 42 patients; 19/42 (45.24%) had migraine with aura, 35/42 (83.33%) had episodic migraine, and 7/42 (16.66%) had chronic migraine.

The demographic and clinical features and the results of psychological assessments and the HPI scores are reported in Table 1.

Overall, 15/42 (35.71% of the patients) had alexithymia: 6 (14.28%) had a definite alexithymia and 9 (21.42%) a borderline degree of alexithymia, according to the TAS-20 score.

**Figure 1:**
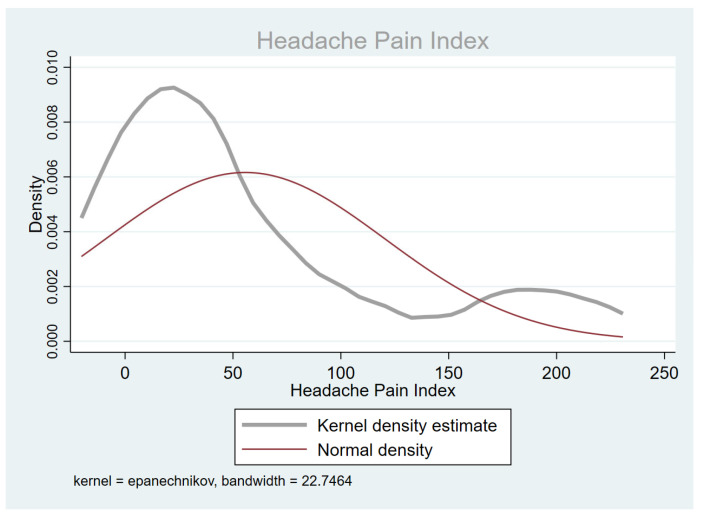
The distribution of the HPI score in the sample.

**Figure 2:**
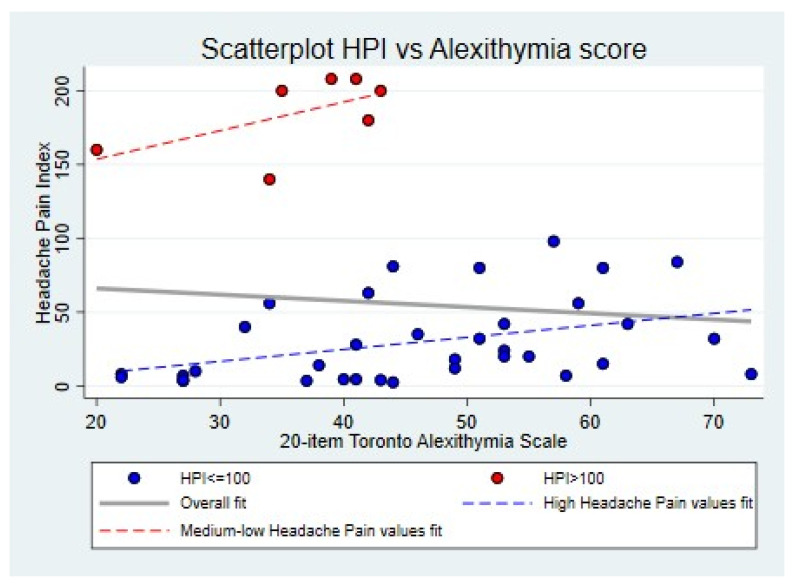
A graphical illustration of the relationship between alexithymia and the HPI score. Note: The scatterplot in Figure 2 illustrates the bimodal distribution described in Figure 1 in an alternative and complementary way. The two distinct subgroups represent the peaks of the curve described in Figure 1 (the one characterized by the highest HPI scores identifies the group that was defined as ‘outliers’). It shows three superimposed regression lines representing exploratory linear fits. The thicker grey line expresses the unadjusted relationship between the TAS and HPI scores in the sample taken as a whole, while the dotted lines represent the same relationship within the two subgroups. Considering the whole sample, the relationship between the TAS-20 and HPI scores is inverse, while the same analysis at the level of each single subgroup suggests that there is a direct relationship.

Figure 1 reports the HPI score distribution in the total sample; the relationship between alexithymia and HPI is illustrated in Figure 2.

The independent variable alexithymia, quantified by the TAS-20 score, in the 42 patients demonstrated an inverse relationship with the dependent variable in-HPI (negative value of the point estimate = −0.005), that is, the higher the degree of alexithymia, the lower the headache pain index, in the absence of statistical significance (*p* = 0.701) (Table 2).

**Figure 3:**
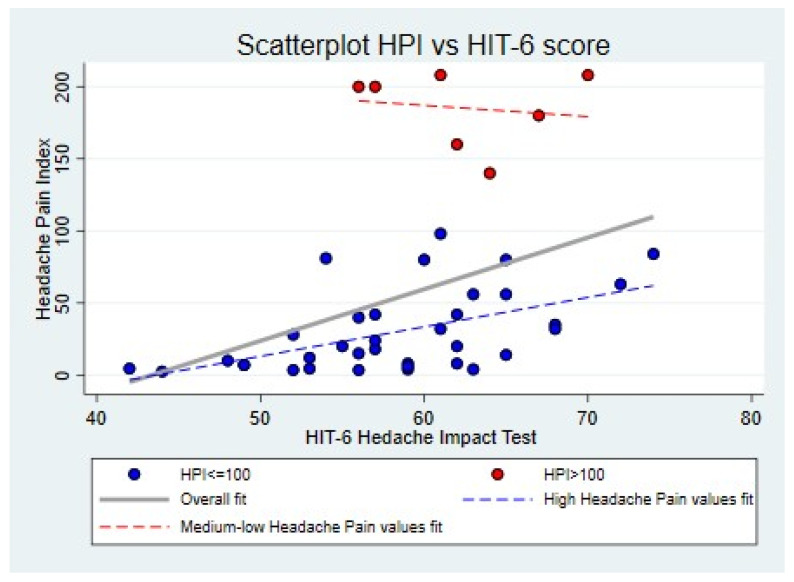
A graphical illustration of the relationship between the alexithymia and the HIT-6 scores.

Figure 1 and Figure 2 evidence the presence of a bimodal distribution of HPI values in the total sample, although this might depend on the effect of chance: due to the design of the “pilot study”, an opportunistic recruitment was made on a small number of cases. However, the distribution of some variables corresponding to important clinical characteristics, such as disease duration, migraine frequency, anxiety, and depression scores, suggests a possibility that a stratum of the sample may actually be representative of a clinically identifiable subgroup. In fact, as shown in the scatter plot in Figure 2, seven patients with an HPI of >100 appeared to be “outliers”, as they had peculiar clinical characteristics that differed from the rest of the sample, that is, a longer duration of migraine disease, a chronic pattern, along with higher scores on the two anxiety (STAI-Y 1 and STAI-Y2) and the depression (BDI-II) scales (Table 1). The same distribution in two distinct subgroups, based on the HPI score (> or ≤100), was also confirmed for the other covariates (HIT-6, see Figure 3). This allowed us to remove this subgroup of patients who differed clinically from the total sample from the final statistical model, as they were characterized by an HPI of >100 and by a more severe psychiatric comorbidity, which may have played a confounding role in the relationship between alexithymia and migraine severity.

It was therefore decided to repeat the analyses exclusively on the remaining 35 patients.

The statistical model on this clinically more homogeneous sample of 35 subjects with an HPI score of ≤100, who were not so influenced by depression and/or anxiety, revealed that the association between alexithymia, expressed as the TAS-20 score, and ln-HPI was not only positive but also statistically significant, consistent with the positive statistically significant value of the logTAS-20 angular coefficient reported in Table 2 (point estimate = 0.032, *p* = 0.010).

**Figure 4:**
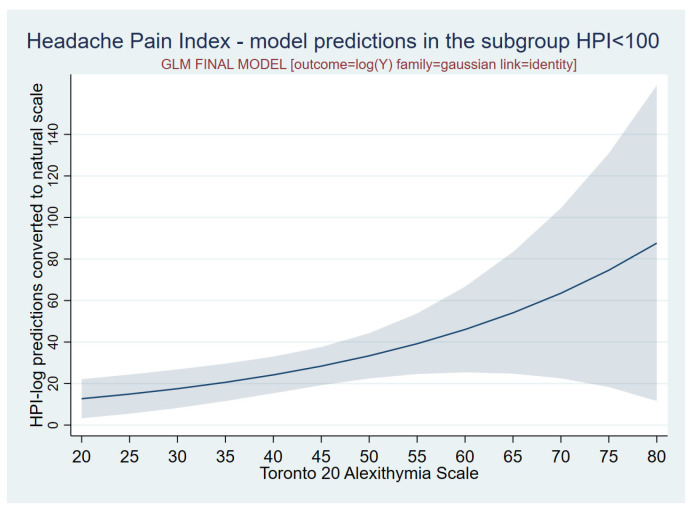
A model prediction of the HPI score using the alexithymia (TAS-20) score in the OLS model (log-normal family = Gaussian link = identity).

The final General Linear Model revealed that the presence of alexithymia, expressed as the TAS-20 score, was predictive of HPI, with a high statistical significance (*p* = 0.010) (Table 2 and Figure 4). In other words, the psychological trait of alexithymia directly affected the frequency of migraine attacks and the pain intensity in this sample, leading us to conclude that the higher the degree of alexithymia, the more severe the migraine phenotype in this pilot study.

Among the other variables, headache-related disability, expressed by the HIT-6 score, was strongly associated with ln-HPI in the subgroup with an HPI score of ≤100 (*p* < 0.0001) (Table 2).

## 4. Discussion

To the best of our knowledge, although preliminarily, this study is the first to suggest that alexithymia plays a significant role in increasing both the frequency and intensity of migraine attacks, expressed by the HPI, whatever the migraine duration.

First, there is a high prevalence of alexithymia in our sample (35.71%), in line with what was reported by Di Tella and Castelli [5] in painful syndromes (about 40%) and, in particular, with Galli et al.’s observation [6] in migraineurs, where defined alexithymic characteristics were found in 35.20% of cases and a borderline level of alexithymia in 14.80%.

As aforementioned, the most relevant finding that came to light during this study was that the higher the degree of alexithymia, the worse the frequency and intensity (expressed as HPI) of migraine attacks. This may well imply that alexithymia is able to affect the clinical pain phenotype of migraine.

These results support the clinical relevance of a multidimensional transnosographic construct in patients suffering from pain disorders, particularly from migraine headaches. The effect alexithymia has in predicting a greater severity of migraine attacks, in terms of frequency and intensity of pain, could hopefully add new scientific evidence to support the emerging role alexithymia plays in pain syndromes, including headache [6,11,12,24].

These findings are consistent with previous ones on the same sample of women affected by migraine, where alexithymia was hypothesized to influence the pain phenotype in migraine, thus increasing the tenderness of the pericranial and cervical muscles, whatever the psychiatric comorbidity [25]. It is also in line with the other literature evidence on the association between alexithymia and somatic symptoms, especially in painful pathologies [5]. Notably, it has been reported that alexithymia is associated with both pain intensity and pain-related functioning in individuals with chronic pain [3,4].

Furthermore, the results of the statistical analysis models show that the disability due to headache, expressed by the HIT-6 score, is strongly associated with the HPI (*p* < 0.0001), confirming how a greater frequency and intensity of migraine attacks correlate with a more serious disabling impact on the quality of life of those affected.

Our finding that alexithymia is not only frequent in female migraineurs but is also able to significantly influence the clinical phenotype of headache, worsening the pattern of pain attacks and aggravating their disabling effect, appears to be in agreement with previous evidence. In fact, the deficient cognitive processing of affective states, which are characteristic of alexithymia, involving the inability to recognize and translate emotions into words and to discriminate between somatic and emotional perceptions, can favor the dysregulation of other biological systems, thus contributing to the development of a physical disease [26], particularly chronic pain syndromes [5].

Furthermore, according to the bio-psycho-social model of migraine [10], the genetic predisposition interacts bidirectionally with other biological and environmental factors in determining the migraine clinical phenotype. This interaction takes place in a complex interplay with psychological factors, including personality traits, traumatic events, and social and lifestyle factors [9]. As it is known that alexithymia is characterized by the inability to “mentalize” emotions, it may be considered a risk factor for the worsening of pain diseases, in this case, migraine. As a matter of fact, the inability to symbolize emotional states and to discern physiological perceptions from emotional states can favor the experience of emotions through somatic means that exclude the usual mental processing. Consequently, alexithymia, within the sphere of migraine pathology, could promote an increase in both the intensity/frequency of headache attacks and the pain perception of pericranial and cervical muscle contraction [25]. All of which increases the disability of this pathology, worsening the quality of life of migraineurs.

Moreover, migraine can be considered a dysfunction of sensory processing in the bio-psycho-social model [10] and is underpinned by a heightened connection between sensory areas and those of the limbic system responsible for the regulation of emotional life and pain processing [10,27]. Notably, the construct of alexithymia has now been expanded to encompass a more complex emotional dysregulation that involves deficits in emotional cognitive processing [28,29]. Neuroimaging studies on alexithymic individuals [30,31,32] have reported structural and functional alterations in brain areas associated with emotional awareness, which include the amygdala, the insula, the anterior cingulate cortex, the fusiform gyrus, and the parahippocampal gyrus. This means that alexithymia may be hypothesized as the expression of an emotional and cognitive dysregulation in migraineurs that is able to enhance the central sensitization of the trigemino-cervical system, consequently increasing both attack frequency and pain intensity, which involves a multifaceted interplay where psychological, biological, and environmental factors are interconnected.

The observation of a relationship between alexithymia and migraine severity suggests, at least from a speculative perspective, that a novel therapeutic approach targeting alexithymia, like cognitive-behavioral therapy, may be able to improve migraine in females, although further studies are needed to support this appealing hypothesis.

We are aware that this study does have some limitations, which could, to some extent, be justified, that is, it is a pilot study and was not calibrated on a sample size calculated beforehand. Although the sample was quite homogeneous, this study included a very small sample of female migraineurs.

The predictive value of alexithymia on HPI emerges from the analysis on the subgroup of 35 women with an HPI of ≤100. The choice not to consider the results obtained on the entire sample, but rather on the larger subgroup of 35 patients with an HPI of ≤100, can be justified by the fact that the two subgroups with an HPI > and ≤100 had remarkable clinically and statistically significant differences in the depression score (BDI-II *p* = 0.037) and in the duration of headache (*p* = 0.0462). Indeed, in the main analysis (on the subgroup with an HPI of ≤100), the psychometric variables (BDI and STAI-I/STAI-II scores) were not included, meaning that it is not possible to rule out the hypothesis that a psychiatric comorbidity may influence the relationship between alexithymia and the HPI score.

A limitation for the generalizability of the study results is that it cannot be stated for sure that the same findings would have been reproduced in a sample of male migraineurs, given the gender differences in migraine pathophysiology.

A further shortcoming of this study is that medication overuse, sleep disturbances, and/or hormonal factors, which could impact migraine severity, were not taken into account. Moreover, only one instrument was used to measure alexithymia, that is, TAS-20. Even if the TAS-20 is currently the best standardized and most validated instrument used for alexithymia research [29] in the assessment of this construct, it would be preferable that further studies adopt a “multi-methodological approach” using multiple tools, such as projective tests, the “observer-rated” Beth Israel Hospital Psychosomatic Questionnaire (BIQ) test [1], or the Toronto Structured Interview for Alexithymia (TSIA) [26].

## 5. Conclusions

Despite the aforementioned limitations, the preliminary results of this pilot study indicate that alexithymia is able to aggravate the clinical expression of migraine in terms of attack frequency and pain intensity, leading to a more debilitating impact on the quality of life of affected patients.

Further studies are welcome to confirm this intriguing link observed between alexithymia and migraine severity and to assess if a novel management targeting alexithymia could improve migraine disability in females.

## Figures and Tables

**Table 1:** The demographical, clinical, and neuropsychological features of the sample (N = 42).

IQR *	Median	(±S.D.) *	Mean	Variables	Subgroups
21.9–54	38.2	16.66	38.89	Age	HPI <= 100
13–17	15	3.55	14.46	Scholarity (years)	N = 35
1–7	3	4.08	4.40	Monthly migraine days	
9–27	15	11.87	18.74	Disease duration (years)	
7–42	20	28.18	29.84	HPI score	
37–57	46	13.69	46.23	TAS-20 score	
7–21	13	9.72	14.54	BDI-II score	
37–53	45	10.43	45.23	STAI-Y1 score	
42–60	51	9.33	51.80	STAI-Y2 score	
53–63	59	7.27	58.23	HIT-6 score	
26.3–52.7	40.8	14.22	41.51	Age	HPI > 100
13–16	14	2.83	14.00	Scholarity (years)	N = 7
20–26	25	3.91	22.57	Monthly migraine days	
18–47	33	14.85	30.86	Disease duration (years)	
160–208	200	26.40	185.14	HPI score	
34–42	39	7.95	36.29	TAS-20 score	
20–27	26	8.56	22.43	BDI-II score	
43–52	49	8.44	49.57	STAI-Y1 score	
46–59	51	6.78	52.43	STAI-Y2 score	
57–67	62	5.06	62.43	HIT-6 score	
22.4–53.9	39.2	16.15	39.32	Age	Total sample
13–16	15	3.41	14.38	Scholarity (years)	N = 42
1–10	4.5	7.94	7.43	Monthly migraine days	
9–30	17	13.04	20.76	Disease duration (years)	
8–80	30	64.75	55.73	HPI score	
35–53	43	13.37	44.57	TAS-20 score	
7–25	15.5	9.90	15.86	BDI-II score	
40–52	45	10.17	45.95	STAI-Y1 Score	
46–60	51	8.89	51.90	STAI-Y2 score	
55–63	59	7.08	58.93	HIT-6 score	

* S.D.: standard deviation. IQR: interquartile range.

**Table 2:** Mutivariate regression results (final GLM, Family: Gaussian, link: identity) dependent variable: ln[HPI score].

Multivariate Regression (General Linear Model–Family: Gaussian, Link: Identity) Outcome = ln[HPI Score]						
Subgroup HPI < 100 (*n* = 35)			Whole Sample (*n* = 42)			Variables
*p*	CI95%	Coefficient	*p*	CI95%	Coefficient	
0.010	0.008 to 0.057	0.032	0.701	−0.032 to 0.021	−0.005	TAS-20 score
0.000	0.034 to 0.119	0.076	0.001	0.038 to 0.139	0.088	HIT-6 score
0.145	−0.007 to 0.048	0.020	-	-	-	Migraine years (n)
-	-	-	0.026	0.005 to 0.079	0.042	BDI-II score
0.010	−6.069 to −0.814	−3.441	0.108	−5.271 to 0.524	−2.374	Intercept

## Data Availability

The data presented in this study are available on request from the corresponding author due to ethical and legal restrictions.
